# Non-invasive multimodal hemodynamic monitoring for vulnerable preterm infants in the transitional period

**DOI:** 10.1038/s41390-025-04303-7

**Published:** 2025-07-22

**Authors:** Lizelle van Wyk, Barend Fourie

**Affiliations:** Dept Paediatrics & Child Health, Stellenbosch University, Cape Town, South Africa

Understanding the complexity of cardiovascular physiology in the transitional period is critical to providing appropriate hemodynamic support for preterm neonates. This period is characterised by a complex interplay of changes in the pulmonary vasculature, systemic vascular resistance, myocardial contractility and compliance and fetal shunts.^1^ Persistence of the fetal circulation is further compounded by any pathological disease process that may present, leading to hemodynamic compromise.^2^

Sick newborns, especially preterm infants, require various interventions during the transitional period, which may negatively affect cardiopulmonary interactions required to maintain a normal cardiovascular function and cerebral blood flow.^3^ Resulting changes in cardiac output (CO), pulmonary and systemic vascular resistance (SVR), cerebral blood flow and end organ perfusion may lead to various morbidities and mortality.^3^ Measurement of these variables may therefore be advantageous in this period to ensure adequate cerebral and tissue perfusion.

Transthoracic echocardiography (TTE) has been used to describe the cardiovascular physiology and pathophysiology occurring in the transitional period.^4^ TTE remains the gold standard in performing cardiac anatomical and functional assessment as well as for diagnosis of congenital cardiac abnormalities. TTE also remains the mainstay of hemodynamic management in neonates in many NICU’s.^5^ Nevertheless, TTE provides a relatively inaccurate assessment of cardiac output (CO) with high inter- and intra-rater variability.^6^ In addition, TTE only provides intermittent hemodynamic assessments.^3^

Continuous hemodynamic variable measurements may provide improved insights into cardiovascular adaptation during the transitional period.^7^ Multi-modal monitoring consists of combinations of a variety of monitors to provide a comprehensive assessment. This includes hemodynamics via thoracic electrical biosensing technology (TEBT), perfusion via near infra-red spectroscopy (NIRS) and perfusion index (PI), cerebral function via amplitude integrated electroencephalography (aEEG) or electroencephalography (EEG) and autonomic function via heart rate variability, alongside routine vital sign monitoring.^8^ In this manner, early hemodynamic perturbations may be detected and interventions may be applied to improve neonatal outcome.^6^

In this issue of the journal, the article by Martini et al., used a multi-modal approach to describe hemodynamic and cerebrovascular consequences in preterm infants, with different degrees of intra-uterine growth restriction (IUGR), as diagnosed by antenatal doppler, during the transitional phase.^9^ The study found distinctive cardiovascular and cerebrovascular patterns specific to certain patterns of Doppler abnormalities in infants during the transitional period.^9^

IUGR is associated with significant neonatal morbidity and mortality.^10^ Suggested pathophysiological mechanisms include glucose intolerance, insulin resistance, catabolite accumulation, and altered amino acid metabolism.^11^ Prenatal cardiovascular affectations of IUGR include vascular and cardiac remodelling as well as global functional impairment.^12^ These abnormalities may persist postnatally, leading to further compromise of an already fragile system. IUGR is also associated with significant long-term sequelae, specifically metabolic syndrome and neurologic sequelae.^13^ With up to 10% of all pregnancies affected by IUGR, this equates to a significant adult morbidity.^10,13^ Early identification and management may therefore mitigate the life-long consequences of this pathology.

The accuracy of TEBT may still require further research.^14^ However, its ability to provide real-time, continuous, longitudinal data without requiring expensive equipment and extensive TTE training makes it suitable for use in many NICUs that may not have access to such equipment and personnel.^14^ Electrical velocimetry (EV), a type of TEBT, was utilised by Martini et al, to continuously monitor hemodynamics for 72 h in IUGR infants.^9^ In infants with abnormal umbilical artery doppler flow but with brain sparing, EV demonstrated increased cardiac contractility, increased CO, increased SVR and impaired cerebrovascular reactivity. This suggested that in-utero fetal compensatory mechanisms persisted into the postnatal transitional period. Progression of fetal compromise to impaired umbilical artery/ductus venosus flow led to increased SVR, decreased stroke volume, decreased CO, decreased PI and persistence of impaired cerebrovascular reactivity, suggesting a failure of compensatory in-utero fetal mechanisms persisting into the neonatal transitional period.^9^ This supports previous observations of prenatal fetal decompensation persisting into postnatal life.^12^ It further shows the value of utilising TEBT, specifically EV, to monitor the hemodynamic status of infants during the transitional period.

NIRS has been recommended for use in critically ill children for hemodynamic monitoring.^15^ NIRS has been used in multiple studies to aid in the determination of hemodynamic compromise in preterm infants.^16–20^ Few studies have used NIRS specifically for hemodynamic monitoring in IUGR infants. Martini et al’s study combined EV, for hemodynamic monitoring, with NIRS, for cerebral perfusion monitoring. Both the brain sparing and impaired umbilical artery/ ductus venosus IUGR infant groups showed impaired cerebrovascular reactivity (reflected by THORx) but normal cerebral tissue oxygenation and oxygen extraction, as measured by NIRS, suggesting maintenance of cerebral blood flow despite compromised cerebrovascular reactivity.^9^ Persistent postnatally impaired cerebrovascular reactivity may lead to impaired cerebral autoregulation, with an increased risk of cerebral hypo- or hyperperfusion.^21^ Cerebral autoregulation is further dependant on blood pressure and CO, which were both non-invasively determined in the current study.

Much research has described single modality monitoring of the cardiovascular consequences of IUGR, but few have used a multi-modal monitoring approach.^22^ Multimodal monitoring has been shown to be feasible for hemodynamic, ventilation and neurological monitoring in preterm infants.^18,23,24^ Multi-modal monitoring may help in identifying infants at additional risk but may also provide opportunities for intervention, whereby the outcome in these infants may be improved.^18^ The possible value of this concept has been demonstrated by Martini et al, in a group of highly vulnerable and diverse IUGR preterm infants.^9^

Various studies using multimodal hemodynamic management have shown improvement in the outcomes of preterm infants.^17,25^ However, these relied on the use of TTE, that only provides intermittent measurements. In lieu of TTE, TEBT may offer an alternative for continuous monitoring of hemodynamic variables. However, this requires more research. Multimodal monitoring would also allow for the monitoring of multiple parameters, allowing for cross correlation of parameters to identify correlation between physiology, such as in Martini et al’s study of the possible effect of IUGR on cerebrovascular reactivity, in lieu of cerebral autoregulation. This would contribute further to the understanding of the arterial remodelling that may occur after severe IUGR.^12^

Multi-modal monitoring per se may not improve neonatal outcomes. Continuous, real-time monitoring of hemodynamic and tissue oxygenation variables may provide valuable insights into the physiology, or pathophysiology, of the transitional period, and other disease processes, offering an opportunity to intervene timeously and improve neonatal outcomes. This may enable early goal-directed therapy in neonates and personalised medicine in neonates, especially in the vulnerable transitional period.^26,27^
